# Intravenous versus intravenous/oral antibiotics for perforated appendicitis in pediatric patients: a systematic review and meta-analysis

**DOI:** 10.1186/s12887-019-1799-6

**Published:** 2019-11-04

**Authors:** Chuan Wang, Yanan Li, Yi Ji

**Affiliations:** Department of Pediatric Surgery, West China Hospital of Sichuan University, #37 Guo-Xue-Xiang, Chengdu, 610041 China

**Keywords:** Oral, Intravenous, Antibiotics, Perforated appendicitis, Complication

## Abstract

**Background:**

The use of oral (PO) antibiotics following a course of certain intravenous (IV) antibiotics is proposed in order to avoid the complications of IV medications and to decrease the cost. However, the efficacy and safety of sequential IV/PO antibiotics is unclear and requires further study.

**Methods:**

The databases, including PubMed, EMBASE and Cochrane Library, were searched. Studies comparing outcomes in patients with perforated appendicitis receiving sequential IV/PO and PO antibiotics therapy were screened. The Newcastle-Ottawa Scale (NOS) and the Jadad score were used to evaluate the quality of the cohort and the randomized controlled portions of the trial, respectively. Statistical heterogeneity was assessed using the *I*^2^ value. A fixed or random-effect model was applied according to the *I*^2^ value.

**Results:**

Five controlled studies including a total of 580 patients were evaluated. The pooled estimates revealed that sequential IV/PO antibiotic therapy did not increase the risk of complications, with a risk ratio (RR) of 0.97 (95% CI 0.51–1.83, *P* = 0.93) for postoperative abscess, 1.04 (95% CI 0.25–4.36, *P* = 0.96) for wound infection and 0.62 (95% CI 0.33–1.16, *P* = 0.13) for readmission.

**Conclusions:**

Our study demonstrates that sequential IV/PO antibiotic therapy is noninferior to IV antibiotic therapy regarding postoperative abscess, wound infection and readmission.

## Introduction

Appendicitis is the most common abdominal condition requiring emergent surgery in the pediatric age group [1, 2]. Perforated appendicitis accounts for 15–50% of cases of pediatric appendicitis [3, 4]. Appendectomy following a course of antibiotic treatment is generally accepted in the practice of managing perforated appendicitis in children. Nevertheless, there is not a consensus regarding the optimal antibiotic regimen in pediatric patients with perforated appendicitis, including the specific antibiotic regimen, treatment duration, and administration route [5, 6]. Peripherally inserted central catheters (PICCs) and intravenous (IV) lines are widely used to administer antibiotics because of the long duration of treatment. Even though a PICC line is more convenient than an IV line, as it can be used after patients are discharged from the hospital, a PICC line still has many of the same disadvantages as an IV line, including activity restrictions, painful insertion, risk of infections and mechanical complications [7–9].

It is suggested here that the use of oral (PO) antibiotics following a course of IV antibiotics could be administered to avoid the complications that may be associated with long-term use of a PICC line for antibiotics. However, it would be concerning if the use of PO antibiotics following an IV antibiotic course increases the risk of complications of perforated appendicitis and results in treatment failure. Although some trials have been conducted to evaluate this problem, those studies failed to draw a robust conclusion due to the small sample sizes of each trail, which ideally would have had a much larger sample size in each treatment arm [5]. We conducted this meta-analysis to answer the question of whether sequential IV/PO antibiotic therapy is equivalent to IV antibiotic therapy.

## Methods

### Study selection

Controlled studies that compared the outcomes of treating with IV antibiotics to the outcomes of treating with a transition to oral antibiotics after appendectomy in patients with perforated appendicitis were included. Perforated appendicitis was defined as a discernable hole in the vermiform appendix or evidence of a perforation such as an extraluminal fecalith in the abdomen. Furthermore, eligible studies were required to record at least one of the following outcomes: postoperative abscess, wound infection or readmission. The eligible studies were limited to those that had been published in English.

### Search strategy

Two researchers (C.W. and Y.J.) independently searched the EMBASE, PubMed and Cochrane Library databases to identify potential studies. The key search terms were ‘intravenous,’ ‘oral,’ ‘antibiotics,’ and ‘appendicitis,’ and these words were combined with the Boolean operator AND. Each of the two investigators independently inspected titles and abstracts and scrutinized full-text manuscripts of the selected studies to identify eligible literature that met the inclusion criteria. Reference lists of eligible literature were reviewed to screen any other potential studies. Based on previous studies, a perforation was defined as a hole in the appendix or a fecalith in the abdomen [10].

### Data extraction and quality assessment

This study was conducted in accordance with the Preferred Reporting Items for Systematic Reviews and Meta-Analyses (PRISMA). Postoperative abscess was defined as the primary outcome. Wound infection and readmission were defined as the secondary outcomes. Two authors (C.W. and Y.J.) independently extracted and recorded the following data from the included studies: the name of the first author, the year of publication, the number of cases and controls, the study design, the primary outcomes, and the secondary outcomes. The Newcastle-Ottawa Scale (NOS) was used to evaluate the quality of the included cohort studies [11]. The total score ranged from 0 to 9, and a study with a score of more than 5 was regarded as a “high quality” study. The Jadad score, ranging from 0 to 5, was used to assess the quality of included randomized controlled trials [12]. Studies with a score of at least 3 were considered to be “high quality” studies.

### Statistical analysis and exploration of heterogeneity

The meta-analysis was conducted by using the Reviewer Manager 5.3 from the Cochrane Collaboration. The Mantel–Haenszel method was used in the meta-analysis. The risk ratio (RR) with 95% confidence intervals (CIs) was employed for the pooled results of all outcomes. The potential for publication bias was assessed using funnel plots. Heterogeneity among studies was evaluated by the *I*^2^ method, with a higher *I*^2^ value indicating a higher heterogeneity. If the *I*^2^ value was less than 50%, a fixed-effects model of analysis was applied; otherwise, a random-effects model was applied.

## Results

**Fig. 1:**
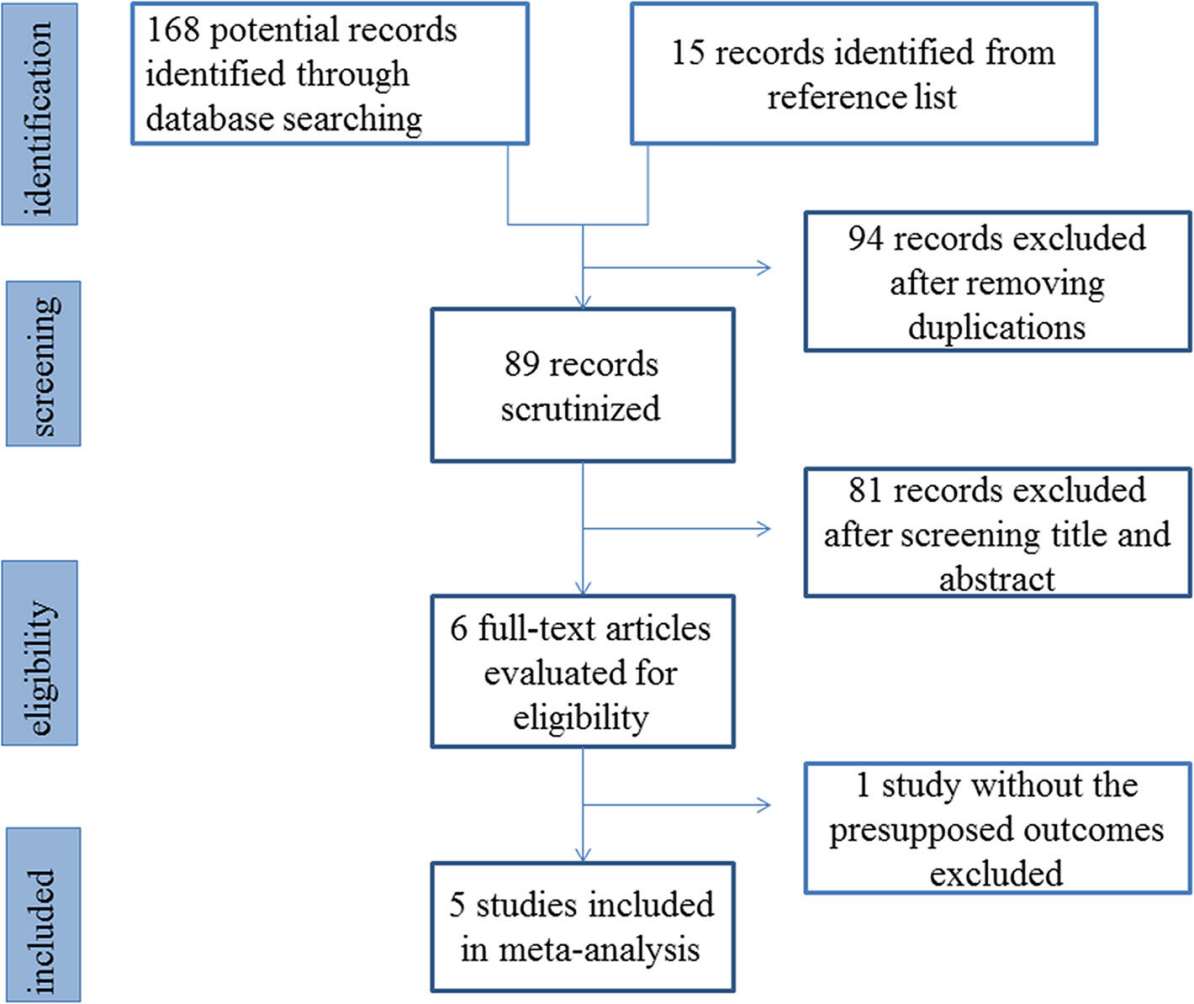
Flow chart of study selection

Figure 1 shows the results of the search and the selection of articles. The low stringency initial screen identified 183 articles through an online search and by reviewing reference lists of relevant publications. Six articles were evaluated for eligibility after further scrutinizing the titles and abstracts. Five studies were included in the final analyses [13–17]. Among the five articles, three were randomized controlled studies and two were retrospective observational studies. Table 1 showed the characteristics and scores of these studies. A total of 580 patients were assigned to the IV group (*n* = 306) or the IV/PO group (*n* = 274). The detailed case numbers of each outcome in the five articles were summarized in Table 2. No obvious publication bias was detected in any of the analyses.

**Table 1:** Characteristics of included studies

Study	Study type	Sample size	Age (years, mean ± SD)	Type of antibiotic	Length of antibiotic therapy (days, mean ± SD)	NOS/JS
Henry E. Rice 2001	RCT	IV/PO: 16	11.9 ± 3.9	IV: Ampicillin & gentamicin sulfate & clindamycin/PO: amoxicillin-clavulanate	10.1 ± 0.5	3
		IV: 10	12.5 ± 3.7	IV:Ampicillin & gentamicin sulfate & clindamycin	10.4 ± 1.3	
Obinna O. Adibe 2008	OCS	IV/PO: 47	9.7 ± 0.52	IV: Ampicillin–Sulbactam & Gentamicin OR Ampicillin–Sulbactam/PO: TMP-SMX & Metronidazole	14 ± 0	8
		IV:102	8.8 ± 0.41	IV:Ampicillin–Sulbactam & Gentamicin OR Ampicillin–Sulbactam	14 ± 0	
Jason D. Fraser 2010	RCT	IV/PO: 50	10.1 ± 4.6	IV: Ceftriaxone & Metronidazole/PO: Amoxicillin-Clavulanate	≥ 5	2
		IV: 52	9.7 ± 4.2	IV:Ceftriaxone & Metronidazole	7 ± 0	
Shannon N. Acker 2016	OCS	IV/PO: 291	9.7 ± 4.1	IV: Ceftriaxone & Metronidazole/PO: NA	NA	8
		IV: 34	8.9 ± 4.5	IV:Ceftriaxone & Metronidazole followed by other types for home antibiotic	NA	
Tara J. Loux 2016	OCS	IV/PO: 123	10.24 ± 4.3	IV: Piperacillin-Tazobactam/PO: TMP-SMX & Metronidazole	15.2 ± 8.4	7
		IV: 98	10.51 ± 4.4	IV:Piperacillin-Tazobactam followed by other types for home antibiotic	15.2 ± 8.5	
Michael R. Arnold 2018	RCT	IV/PO: 38	10.1 ± 3.6	IV: Ertapenem/PO: Amoxicillin-clavulanate	10 ± 0	3
		IV: 44	12.3 ± 3.6	IV:Ertapenem	10 ± 0	

*RCT*: Randomized controlled trial; *OCS*: Observational clinical study; *IV*: Intravenous; *PO*: Oral; *NA*: Not available; *NOS*: Newcastle-Ottawa Scale score; *JS*: Jadad score

**Table 2:** Summary of the outcomes of included studies

Study	Sample size	Postoperative abscess	Wound infection	Readmission
Henry E. Rice 2001	IV/PO: 16	0 (0%)	1 (6%)	NA
	IV: 10	0 (0%)	1 (10%)	NA
Obinna O. Adibe 2008	IV/PO: 47	2 (4.2%)	0 (0%)	NA
	IV: 102	2 (2%)	2 (2%)	NA
Jason D. Fraser 2010	IV/PO: 50	10 (20%)	NA	NA
	IV: 52	10 (19%)	NA	NA
Shannon N. Acker 2016	IV/PO: 291	11 (3.8%)	NA	44 (15.1%)
	IV: 34	1 (2.9%)	NA	6 (17.6%)
Tara J. Loux 2016	IV/PO: 123	NA	NA	19 (15.4%)
	IV: 98	NA	NA	8 (8.1%)
Michael R. Arnold 2018	IV/PO: 38	3 (7.9%)	2 (5.3%)	6 (15.8%)
	IV: 44	5 (11.4%)	1 (2.3%)	6 (13.6%)

*IV*: Intravenous; *PO*: Oral; *NA*: not available

### Postoperative abscess

**Fig. 2:**
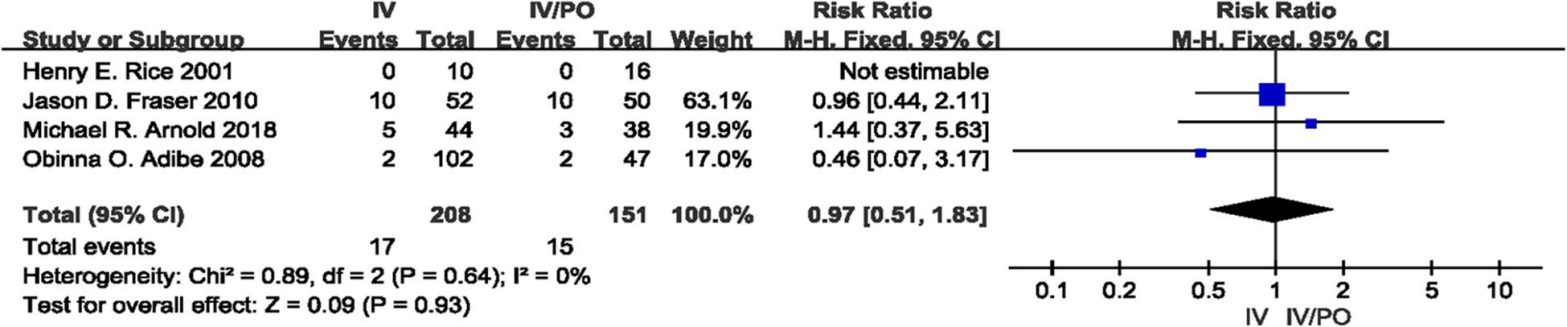
Forest Plot showing the risk ratio for the occurrence of postoperative abscess in the intravenous/oral and intravenous groups

Four studies investigated the occurrence of postoperative abscess in pediatric patients with perforated appendicitis [13–15, 17]. The occurrence rate of postoperative abscess was 8.2% (17, *n* = 208) in the IV group and 9.9% (15, *n* = 151) in the IV/PO group. There was no discernible heterogeneity among the four studies (*I*^2^ = 0%). The pooled RR was 0.97 (95% CI 0.51–1.83, *P* = 0.93). The results showed that there was no significant difference in the occurrence rates of postoperative abscess between the two groups (Fig. 2).

### Wound infection

**Fig. 3:**
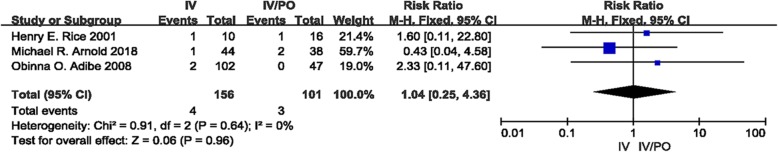
Forest Plot showing the risk ratio for the occurrence of wound infection in the intravenous/oral and intravenous groups

Three studies reported wound infections [14, 15, 17]. In total, wound infection developed in 4 of 156 patients in the IV group and 3 of 101 patients in the IV/PO group. No heterogeneity was identified among these studies (*I*^2^ = 0%). Our meta-analysis revealed that there was no statistically significant discrepancy between the two groups (RR 1.04, 95% CI 0.25–4.36; *P* = 0.96) (Fig. 3).

### Readmission

**Fig. 4:**
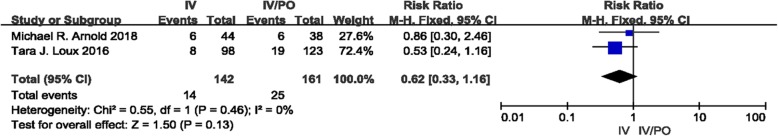
Forest Plot showing the risk ratio for the occurrence of readmission in the intravenous/oral and intravenous groups

Readmission was reported in two studies [14, 16]. The *I*^2^ method detected no significant heterogeneity with *I*^2^ = 0%. There was no statistically significant discrepancy between the two groups regarding readmission rates, with a pooled RR of 0.62 (95% CI 0.33–1.16; *P* = 0.13) (Fig. 4).

## Discussion

Perforated appendicitis is a common abdominal emergency in children. Antibiotic therapy combined with appendectomy is used worldwide to treat perforated appendicitis. In 1994, Lund et al. [18] proposed a “gold standard” of antibiotic therapy for perforated appendicitis. The treatment regimen included 10 days of intravenous antibiotics. However, the IV route results in higher costs, longer length of hospital stay and less safety than the PO route. Thus, the conversion to PO after a course of IV antibiotics was proposed as an alternate to IV-only treatment. Several studies, including randomized controlled studies and observational studies, were conducted to examine this option [13–17, 19–21]. Recent studies provide evidence that PO antibiotic treatment was at least as effective as continuing IV antibiotic therapy. In a prospective study including 80 children with perforated appendicitis, the investigators provided satisfactory results that after appendectomy patients can be safely discharged home with a 7-day course of PO antibiotics when enteral intake is tolerated, regardless of the presence of fever or leukocytosis [22]. Although the outcomes showed that the conversion to PO after a course of IV antibiotics seemed to be feasible, a robust conclusion could not be drawn due to the small sample sizes in these studies.

Postoperative abscess is a main complication of perforated appendicitis and developed in approximately 12% of the patients with perforated appendicitis [23]. Postoperative antibiotic therapy can decrease the risk of abscess [24], and the optimal administration route is usually thought to be IV. In this meta-analysis, the result indicates that the IV/PO route is as effective as the IV route in terms of preventing an abscess. Moreover, it has been reported that PO antibiotics treating already-formed abscesses achieved equivalent outcomes as IV antibiotics [21]. Thus, conversion to PO may be initiated when the patient tolerates an oral diet.

Wound infection is also a common complication of perforated appendicitis, with a prevalence of approximately 17% [23]. Previously, some investigators suggested that wounds should be left open in the presence of perforated appendicitis to avoid an increased likelihood of wound infection and longer hospital stay and cost [25, 26]. A growing number of studies indicated that primary wound closure after appendectomy would be safe even in cases of perforated or gangrenous appendicitis [27–29]. In addition, wound infections may also be reduced by using antibiotic therapy. Our study shows that the use of IV/PO antibiotics is noninferior to IV antibiotics with regard to wound infection. In the past, intravenous injection of antibiotics was widely used to treat infectious disease due to a lack of appropriate oral antibiotics. With the development of medical therapy, more broad-spectrum oral antibiotics have been created. Thus, more studies should be conducted to investigate whether PO antibiotics could be used to treat reflux cholangitis or other severe infectious diseases that require long courses of antibiotics.

Readmission is mainly attributed to infection, wound complications and small bowel obstruction. In this study, we found that IV/PO antibiotic therapy did not increase the risk of readmission compared with IV antibiotic therapy. The use of IV-only actually increased the risk of readmission compared with PO in patients with complicated appendicitis, and it lead to more repeat visits due to complications associated with the IV or PICC line [20]. Although the introduction of PICC provided a therapeutic advance, as it allowed patients to be discharge home to complete their IV antibiotic therapy once recovered from their operation, the use of PICC is not without risk. Well-known adverse events, including painful insertion, activity restrictions, and risk of mechanical and infectious complications, were commonly documented in patients using PICC lines [8]. In patients receiving PO antibiotics, the PICC complications can be avoided entirely. Only a small number of patients experience a failure of PO antibiotics, and this is primarily due to protracted vomiting. Our study suggested that enough bioavailability and blood concentration of antibiotics could be achieved to treat severe infectious disease by the PO route when the gastrointestinal function recovered.

Some limitations of this study should be recognized. The included studies are limited, and further studies should be conducted to investigate this issue. The regimens of IV/PO antibiotics and IV antibiotics vary among studies because there is not yet a consensus formed regarding an optimal antibiotics option. The timing for conversion from IV to PO is also different among the selected studies.

## Conclusions

This research provides valuable evidence with respect to the efficacy and safety of sequential IV/PO antibiotic therapy in patients with perforated appendicitis. Our study demonstrates that sequential IV/PO antibiotic therapy is equivalent to IV antibiotic therapy regarding postoperative abscess, wound infection and readmission. Further studies should be conducted to confirm this conclusion and the optimal timing for conversion.

## Data Availability

The datasets analysed during the current study are available from the corresponding author on reasonable request.

## Abbreviations

CI: Confidence interval
IV: Intravenous
NOS: Newcastle-Ottawa Scale
PICC: Peripherally inserted central catheter
PO: Oral
RR: Risk ratio
